# Mr. Tod on the Anatomy and Physiology of the Organ of Hearing

**Published:** 1832-10-01

**Authors:** 


### XIII.

**The Anatomy and Physiology of the Organ of Hearing; with Remarks on Congenital Deafness, the Diseases of the Ear, some Imperfections of the Organ of Speech, and the proper Treatment of these several Affections. By *David Tod,* Member of the Royal College of Surgeons. Octavo, pp. 147. London, 1832.**

It has long been a matter of complaint that our knowledge of the diseases of the ear is limited and imperfect. The cause is to be found in the intricate arrangement of the organ itself, and the difficulty of procuring accurate and satisfactory examinations of its condition after death. The cultivation of morbid anatomy which has done so much towards advancing our knowledge of the diseases of other parts, has contributed but little to the increase of our information or the improvement of our treatment in acoustic surgery. Mr. Tod, the author of the little work before us, appears to have engaged with much zeal in the investigation of the healthy structure and physiology of the organ of hearing. As such researches do not come within the scope of a medical review, we must content ourselves with recommending such of our readers as take an interest in anatomical questions to peruse the work itself. We can merely notice some remarks on the diseases of the ear.

Mr. Tod gives some cases of fungus of the meatus which were cured, as they generally are, by removal of the fungus and the subsequent application of the lunar caustic. We have seen many instances of this description, and, so far as we recollect, all yielded to the application of the lunar caustic, with occasional touches of the kali purum. Mr. Tod observes that he has often witnessed erysipelatous inflammation of the auricle and appendix, and has found it a difficult disease to treat, as well as a common one. His plan appears to consist in attention to the bowels and digestive organs, and an open blister to the nape of the neck. He has frequently found it necessary to continue all the remedies, especially the blister, for twelve and eighteen months after every symptom had apparently ceased. We suspect that many patients would rebel against such discipline. The following is deserving of extraction.

"Mr. B., ætat. 30, of a delicate constitution, consulted me on account of being troubled with various kinds of noises in his ears. These he told me were always more severe during the night when in bed, than during the day, and never ceased for more than five minutes at a time. He informed me further, that when the noises ceased, they did so suddenly, and returned in like manner; that when he swallowed his saliva or yawned, he either stopped the noise in one or both ears for a few minutes, or changed its character; and that the sounds were often so loud, that he could not hear when a cart or carriage was driving past him. On examining the Meatus of each Ear, there did not appear the least secretion of wax,—but nothing else of a disordered nature could be discovered.

Considering the phenomena which he complained of to be owing to this want of cerumen, and to some faulty function of the secreting vessels of the Labyrinth and Tympanum, producing a sympathetic constriction of the Eustachian Tubes, I directed him to take five grains of blue pill, combined with a quarter of a grain of opium, three times a day, until his gums became affected; to keep the Meatus of each Ear continually plugged with lint moistened in linimentum camphoræ cum liq. volat. c. c., and to apply a perpetual blister to the nape of the neck.

In the course of a week he appeared a little, but not to any great extent, better.

He complained of the pills producing great pain in his bowels, and of his mouth being a little sore. I ordered the pills to be discontinued; and as his mouth did not appear to be much affected, directed him, instead, to rub in two drachms of the unguent. hydr. fort. every night. I also punctured the drum of each Ear, and recommended the other remedies to be continued.

In the course of another week his mouth became affected, but the noises in the Ears still annoyed him much, although greatly diminished.—I discontinued the ointment, and prescribed a little aperient medicine, combined with infus. rosæ, and sulph. quininæ, three times a day.

In the course of ten days his mouth got nearly well, his general health became much improved, and the noises in the Ears had almost entirely ceased. Ordered him to continue the quinine, to omit the aperient, and to take five grains of blue pill every night for a week, then every other night for a month. At the expiration of this period his general health seemed to have undergone a complete revolution, and his hearing became quite restored." 122.

In another similar case, a similar plan of treatment ultimately failed. We have only room for one more passage; it exhibits Mr. Tod's mode of introducing a bougie into the Eustachian Tube.

"After selecting a bougie of the calibre and shape of the tube, and oiling its extremity, I ascertain by external measurement the distance from the orifice of the nose to the Cavitas Tympani, and then mark the instrument so as to know when I have succeeded in reaching the posterior extremity of the canal: I then bend the point a little, and pass it along the floor of the nose, with the point touching its outer and under surface, to the part at which the membrane of the nose is about to ascend to form the external wall of the cavity, until I have reached the fauces: I then gradually raise the point of the bougie, so as to touch the lateral surface of the faucial cavity as I push it backwards, turning it in its course first a little to one side and then to the other; and when I find that it becomes loosely fixed, and does not descend towards the pharynx, I conclude that it has entered the Eustachian Tube: I then continue pressing the bougie gently onwards until I perceive the mark which I had made has reached the orifice of the nose; and after allowing it to remain a few minutes, withdraw it in as gentle a manner as it was introduced. When, however, I find that the point of the instrument does not get into the faucial extremity of the Tube, I make it a rule to lay it aside for two or three weeks, and to employ a silver probe instead. But sometimes the passing of the latter instrument will be attended with as much difficulty as the former; and when this is the case, the same rule should be observed regarding its repetition: for the principles upon which these instruments should be employed are the same as those which ought to guide us in the treatment of susceptible surfaces generally. After subduing the action which has been excited in the diseased part by the means recommended, the instruments should again, but not till then, be employed. The first application of the bougie will in general be accompanied with some pain; but as this sensation does not arise from the presence of any additional morbid cause, it will in a short time subside, and the patient will feel considerable relief. We must not, however, repeat the stimulus too frequently, for by passing these instruments too often, as much harm as good may be done. In treating obstructions of the Eustachian Tubes, we should be guided by the same principles which serve us in the cure of other diseases. When we repeat a stimulus too frequently, we bring on considerable irritation,—and we ought to recollect that irritation was the original cause of the disease. No instrument should be introduced for some weeks after the patient has applied for relief; for this will always be attempted too soon, unless we have succeeded in soothing the irritation by exciting secretion: even after we have begun the use of instruments, once or twice a week should be the extent of our visitations. By introducing the instruments oftener, 'we have,' to use the words of the late Mr. Abernethy, 'not only to counteract the irritation which previously existed, but the irritation which we have ourselves produced.' And it is from a want of attention to this circumstance, that the operation which we have been discussing has so repeatedly proved unsuccessful." 131.

We repeat that this little work evinces zeal, industry, and perseverance on the part of Mr. Tod.

